# Laser-Controlled Propulsion of a Microbubble Rolling on a Carbon Nanocoil Rail

**DOI:** 10.3390/nano16010005

**Published:** 2025-12-19

**Authors:** Yuli Liu, Si Li, Yanming Sun, Jinlu Li, Yuanyong Dai, Mengmeng Zhang, Jian Shen, Lujun Pan

**Affiliations:** 1 Fundamental Education Department, Dalian Neusoft University of Information, Dalian 116023, China; liuyuli@neusoft.edu.cn (Y.L.); 2321350883@dnui.edu.cn (J.L.); 13387886754@163.com (Y.D.); 2 School of Foreign Languages, Dalian Neusoft University of Information, Dalian 116023, China; lisi@neusoft.edu.cn; 3 College of Physics and Optoelectronic Engineering, Shenzhen University, Shenzhen 518060, China; sunym@szu.edu.cn; 4 Department of Criminal Technology, Liaoning Police College, Dalian 116036, China; ala93fly@hotmail.com; 5 School of Physics, Dalian University of Technology, Dalian 116024, China

**Keywords:** laser irradiation, CNC rail, microbubble, laser-controlled, propulsion, rolling, movement

## Abstract

Controllably propelling microbubbles in microchannels within a microfluidic chip is of great scientific significance yet remains challenging. In this work, we employ carbon nanocoils (CNCs) as a laser-energized rail for propelling microbubbles to the desired position on the inner sidewall of microchannels by laser irradiation at the liquid-CNC interface. Laser-controlled microbubbles can be generated, transported to a desired location, stopped, and re-mobilized repeatedly without a significant change in volume on the microchannel within a microfluidic chip by controlling the laser spot. The microbubbles exhibit a rolling motion at the liquid-CNC interface due to stronger convectional flow induced by a dynamic, mobile thermal gradient generated by a scanning laser spot. The photothermal conversion properties and hydrophobic surface of the CNCs enable the CNCs to function as a laser-energized rail for microbubble propulsion. These results demonstrate that laser-controlled microbubbles rolling on CNC rails have good mobility and can be accurately manipulated in a microchannel chip. This approach leverages a dynamic thermal gradient, departing from static control methods to enable on-demand, reconfigurable manipulation of microbubbles, which opens up new possibilities for lab-on-a-chip and microfluidic applications.

## 1. Introduction

The investigation of microbubbles transport in microfluidic chips represents a significant research direction at the intersection of microfluidics and micro/nanoscale manipulation [1,2,3]. Currently, the formation and transport of microbubbles in liquids have attracted significant research interest across multiple fields, owing to their small size, large specific surface area, high adsorption efficiency, and slow rising velocity in water. These unique properties underpin their promise in diverse applications [4,5,6]. In biomedicine and targeted therapy, drug molecules or gene fragments can be attached to microbubble shells. These loaded microbubbles can be precisely driven to lesion sites, such as tumors, by external energy fields and then ruptured using ultrasound to release their payload locally, thereby enhancing drug efficacy and enabling precision medicine [7,8]. In microfluidics and lab automation, microbubbles serve as versatile functional units. The motive force generated by bubbles within microfluidic channels allows them to act as microrobots or micro-actuators. Their controlled manipulation enables the pushing, capturing, and separation of particles (e.g., cells, bacteria) on chips [9,10]. Consequently, investigating the motion and transport of microbubbles is of considerable scientific and technological importance for a range of cutting-edge applications, including microrobotic actuation, targeted drug delivery, micro-assembly, and localized material synthesis [11,12].

Traditional methods for microbubble manipulation primarily rely on external injection and fluid-driven passive transport [13,14,15], which often suffer from limited precision and instability under fluctuating flow conditions, making them inadequate for high-precision micro-operations. In recent years, advances in micro-nanofabrication and precise control technologies have facilitated novel approaches for refined bubble manipulation [16,17], such as optoelectronic tweezers, optical tweezers, and dielectrophoresis [18,19,20,21]. Among these, techniques based on thermocapillary effect have shown promising potential [22,23,24]. Thermal gradients have been utilized to drive fluids and objects at the microscale and nanoscale, with applications in laser cell surgery, fluid mixing, and microchannel pumping. The principle of the thermocapillary effect relies on temperature gradient-induced changes in interfacial tension, which subsequently generate Marangoni flow [25,26]. This flow can effectively drive the microbubbles to move in a controllable manner within microchannels [27,28]. Such methods typically require an external energy field to establish the temperature gradient. Common approaches to generating temperature gradients include resistive heating [29], direct illumination of the liquid [30], and light absorption by a substrate [31]. Among these, light-induced heating has attracted significant academic interest due to its experimental simplicity and ease of operation, exhibiting advantages such as non-contact operation, fast response time, high safety, ease of application, as well as wireless and remote manipulation capabilities [32,33,34,35]. Ivanova and co-workers reported that the use of photo-absorbing liquids as photothermal conversion media enables the generation of temperature gradients, thereby achieving guided motion of micrometer-sized bubbles within the light-absorbing solution [36]. Aaron T. Ohta and co-workers reported that the motion of air microbubbles in liquid can be actuated via optical illumination of an absorbing substrate, independent of the optical properties of the liquid. The amorphous silicon substrate, which acts as an absorbing substrate, converts optical illumination into a thermal gradient to drive the movement of air microbubbles [37]. Liguo Dai and co-workers reported that the metallic titanium film is sputtered onto a glass sheet to absorb the laser beam for the generation and movement of microbubbles in a silicone oil medium [31].

However, the liquid introduced into microchannel chip and the absorption layer deposited on the chip substrate, both of which exhibit optical absorption properties coupled with high photothermal conversion efficiency, attenuate transmitted light and reduce penetration efficiency at the bottom. Furthermore, microbubbles are confined to motion solely on the bottom absorbing substrate rather than in mid-channel, thereby significantly limiting their maneuverability. The requirement for high optical transparency in applications such as absorbance detection and three-dimensional high-resolution imaging imposes strict constraints on the chip material. Therefore, there is a lack of research on the precise motion control of microbubbles in the microchannel chip without affecting the optical transparency of the microchannel.

Among various light-absorbing materials, carbon nanocoils as quasi-one-dimensional nanomaterials have attracted considerable research interest in recent years due to their exceptional photothermal properties. The nanostructures of CNCs exhibit remarkable broadband optical absorption capabilities, enabling efficient light capture across a wide spectrum [38]. The high thermal conductivity of CNCs facilitates rapid conversion of absorbed photons into thermal energy and effective heat dissipation [39,40]. Furthermore, the unique helical morphology of CNCs contributes to these distinctive characteristics, making them particularly promising for advanced photothermal applications [41,42,43,44,45,46,47]. In combination with hydrophobic characteristic of the CNC’s surface, the CNC surface can effectively trap and stabilize microbubbles in an aqueous environment [48,49,50].

In this study, a novel method is proposed for the controllable generation and precise propulsion of microbubbles along predefined CNC rails, which is realized by laser irradiation at the interface between liquid and CNC to cause dynamic thermal gradient. A relative motion of the heating source (laser spot) will develop asymmetric thermal distribution across the heating surface (CNCs), leading to a propulsive force for microbubble movement. Laser-induced vapor microbubbles exhibit the forward rolling motion at the liquid-CNC rail interface and smoothly follow the path of the laser spot, indicating that the light control method is capable of guiding precise movement of a microbubble along any complex trajectories in a 2D plane. In addition, CNC rails can be integrated with the inner sidewalls of a microfluidic chip, which does not compromise the optical transparency of the top and bottom substrates in the microfluidic chip. This method integrates a mobile heating source with a multifunctional CNCs (characterized by its efficient photothermal conversion, stable hydrophobicity, and unique helical structure) to successfully construct a laser-energized CNC rail, which enables the on-demand generation and instantaneous transport of microbubbles on microchannel sidewalls. Overall, this novel rolling-based propulsion method for moving microbubbles within a microfluidic chip exhibits the characteristics of non-physical contact, fast response time, and precise motion control.

## 2. Materials and Methods

### 2.1. Preparation of a Paraffin/Carbon Nanocoil Mixed Sample

**Figure 1:**
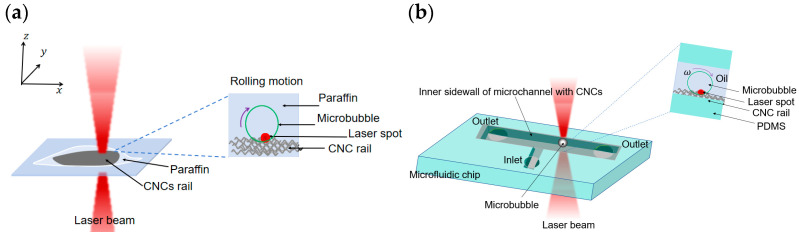
(**a**) Schematic diagram of a vapor microbubble generation and movement in paraffin medium under a laser irradiation. (**b**) Schematic diagram of a vapor microbubble generation and movement in soybean oil medium in a microchannel of a microfluidic chip. A linearly polarized laser diode (λ=1064 nm) with a tunable output power (0−3 W) is used as the light source. The diameter of laser beam spot is approximately 4 μm. The bubble generation process is recorded using a charge-coupled device (CCD) camera system.

The CNCs used in this experiment were synthesized via chemical vapor deposition (CVD) [51,52]. Using C_2_H_2_ as the carbon source, the reaction was conducted at 710 °C for 2 h, resulting in the formation of a large quantity of CNCs on a Si substrate. A mixed solution of Fe_2_(SO_4_)_3_/SnCl_2_ was employed as the catalyst precursor for the preparation of the carbon nanocoils. The carbon nanocoils, after being peeled off from the substrate, exhibited a length of approximately 5–10 μm, a coil diameter of about 500 nm, an average fiber diameter of around 250 nm, and a pitch of roughly 300 nm. A medical crystalline wax flake (mass 0.1 mg) was placed beneath the carbon nanocoil cluster flake (mass 0.02 mg) to form a paraffin/carbon nanocoil mixed sample, which was then positioned on a coverslip in the optical tweezers setup. After the laser (optical tweezers system, LOT II, ZHONGKE Optical Tweezers Technology Co., Ltd, Beijing, China) was focused on the carbon nanocoil, the paraffin melted and completely encapsulated the carbon nanocoil as shown in Figure 1a. The top-right corner of Figure 1a shows a schematic diagram of the laser irradiation at the paraffin-CNC interface.

### 2.2. The Design of Microfluidic Chip

CNCs clusters were dispersed in deionized water (DI water) and subjected to ultrasonication to prepare a CNCs aqueous solution. The dispersed CNCs were injected into the rectangular channel of a microfluidic chip (GH-LD-20, CChip scientific instrument Co., Ltd., Suzhou, China) using a syringe pump (LSP01-2A, Longer Pump, Halma, Bucks, England) [43]. The microfluidic chip used in the experiments was primarily made of polydimethylsiloxane (PDMS). The rectangular microchannel in the PDMS chip had a height of 20 mm and a width ranging from 20 to 100 mm. During the injection process with the syringe pump, the combined effects of hydraulic pressure and CNC adsorption enabled the dispersed CNCs to attach to the inner walls of the microchannel, forming CNC rails. As schematically shown in Figure 1b, a microbubble was optically guided to roll along the CNC rails that are attached to the inner wall of an oil-filled microchannel. The top-right corner of Figure 1b displays a planar view of the laser irradiation on the oil-CNC rail.

## 3. Results

### 3.1. Laser-Controlled Microbubble Roiling Motion on CNC Rails

**Figure 2:**
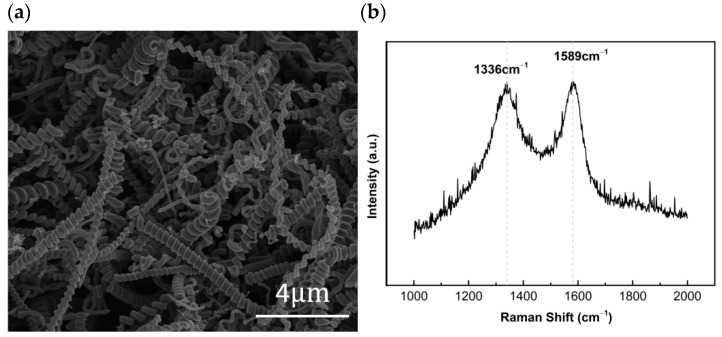
(**a**) Scanning electron microscope image of CNCs. (**b**) Raman spectrum of CNCs [44].

The fabricated CNCs are characterized by a scanning electron microscope (SEM) and a Raman spectrometer (lnvia, Renishaw, Gloucestershire, UK). The SEM image displays the unique helical structure of CNCs (Figure 2a). The Raman spectrum of the CNCs indicates a D band at 1336 cm^−1^ and a G band at 1589 cm^−1^, which represent the defects in the lattice and the Sp^2^ hybridized in-plane stretching vibration of carbon atoms, respectively, as shown in Figure 2b [44].

**Figure 3:**
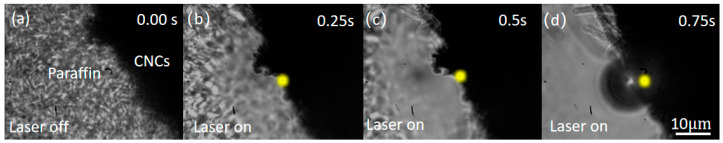
(**a**–**d**) Generation of a vapor microbubble on the CNC rails immersed in molten paraffin. The yellow areas represent the position of the laser spot.

As discussed in previous studies [53], the CNCs exhibit high near-infrared absorption efficiency and extremely low thermal conductivity, resulting in significant localized heat accumulation. Figure 3 shows that when the laser beam is focused at CNC-paraffin interface, localized heating causes the paraffin to melt in the irradiated zone over time. Then, a vapor microbubble is created at the CNC-paraffin interface in the melted paraffin solution following irradiation for 0.75 s. Moreover, the microbubble grows as the laser irradiation time increases and gradually shrinks after the laser is turned off. This phenomenon is very similar to what we observed in previous experiments conducted in water [53]. It is found that no microbubble generation or noticeable change in the paraffin occurs when the laser is focused on pure paraffin (without CNCs) under the same power condition.

**Figure 4:**
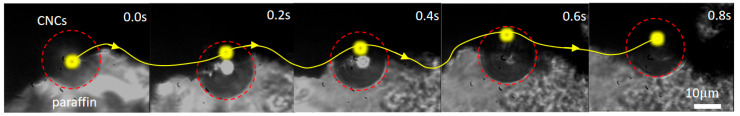
Stack of images (inter-frame interval: 0.2 s) showing a 20-μm-diameter microbubble rolling forward on CNC rails at a speed of about 18 μm/s, guided by a laser beam (laser power of 0.78 mW) in molten paraffin. The yellow areas and line indicate the laser spot position and trajectory, respectively.

However, when the laser spot is moved along the interface, a temperature gradient is established at the paraffin-CNC interface. Figure 4 shows an optically propelled microbubble with a diameter of 20 μm tracking the motion of the laser spot at the paraffin-CNC interface under a laser power of 0.78 mW. The microbubble rolls in tight contact with the paraffin-CNC interface by manually adjusting the motion position of the focusing spot, leading to a movement at the speed of approximately 18 μm/s (also see Video S1, Supporting Information). Increasing the moving speed of the laser spot, the microbubble maintains stable trajectory tracking with the laser spot, never deflects significantly, and remains unchanged in size during movement. When the laser spot deviates from the CNC rail, the microbubble stops rolling instantly, gradually shrinks in size, and finally disappears. Owing to the hydrophobicity of CNCs, the CNC rails effectively capture and stabilize microbubbles in aqueous environments. Thus, The CNC rails not only provide a stable supporting substrate but also maintain and control a rolling motion for the microbubbles via the rolling friction generated at the bubble-rails interface. The laser-controlled propulsion of microbubbles on CNC rails exhibits stability and high efficiency.

**Figure 5:**
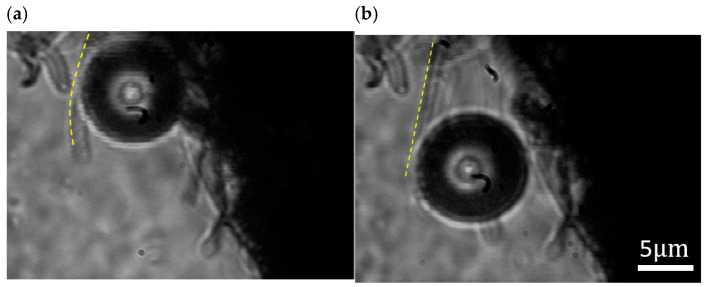
A microbubble rolling forward on a single CNC rail under laser guidance (**a**) before and (**b**) after laser-controlled movement in molten paraffin. The yellow dashed line indicates the outline of the single CNC rail.

To further explore the feasibility of using CNCs as transport rails, a single CNC serving as a guiding rail for microbubble motion is also investigated. Figure 5 shows that an optically propelled microbubble moves along a single CNC following a laser spot.

**Figure 6:**
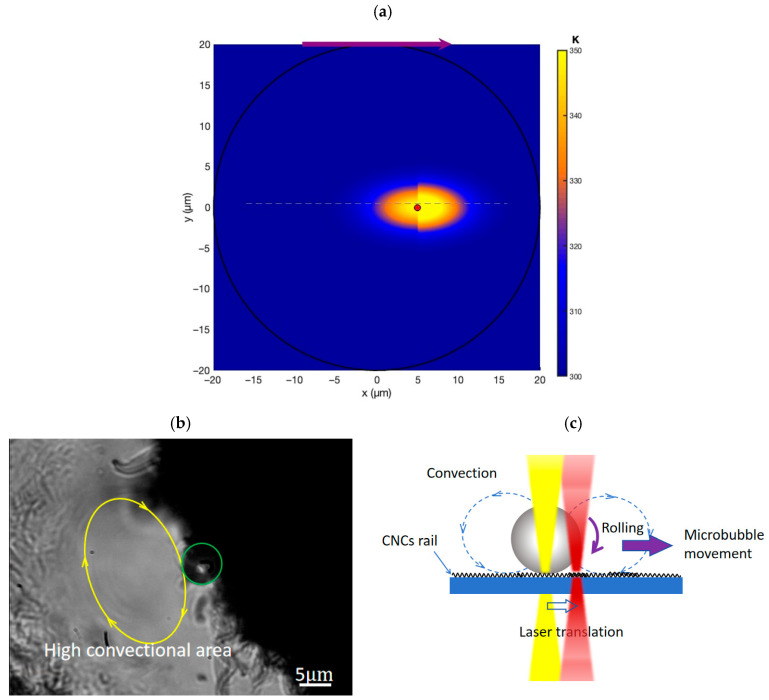
(**a**) Simulation of the dynamic temperature gradient induced by a moving laser beam irradiation on a CNC rail. The red dot indicates the position of the laser spot. The purple arrow indicates the direction of laser movement along the CNC rail. (**b**) Visualization of convectional flow using 2 μm carbon microparticles in DI water. The yellow arrows indicate the high convection around the laser spot. The green circle represents microbubbles. (**c**) Schematic diagram of a microbubble rolling under the influence of flow field.

The ability of thermophoretic convection generated by local heating to act as a driving force has been extensively investigated [54,55]. The dynamic temperature gradient is numerically simulated using MATLAB (version R2020b Update 2, The MathWorks, Natick, MA, USA). Figure 6a displays the simulation result induced by a moving laser beam irradiating on a CNC rail. To confirm the generation of strong convective flows around a microbubble, the sample is immersed in deionized water by droplet deposition on the sample surface and carbon particles with a size of 1 μm are dispersed in an aqueous solution. Figure 6b (also see Video 2, Supporting Information) exhibits the high-convection area around the light-induced microbubble, as revealed by tracking particle trajectories near the heat source. When moving the laser spot along the paraffin-CNC interface, the relative motion of the heating source along the paraffin-CNC interface induces asymmetric thermal distribution across the interface, leading to an asymmetric temperature gradient around a microbubble. Consequently, an asymmetric flow field develops, generating a stronger convective flow in the direction of the laser movement, leading to a driving torque acting on the microbubble (Figure 6c). Additionally, due to the excellent hydrophobicity of the CNC’s surface, the small contact area between the microbubble and the surface of CNC rail is beneficial to the reduction in frictional torque [45,49,50]. Under the continuous action of the driving torque and frictional torque, the bubble maintains the rolling motion, thereby achieving directional movement.

### 3.2. Laser-Controlled Microbubble Roiling Motion Within a Microchannel Chip

**Figure 7:**
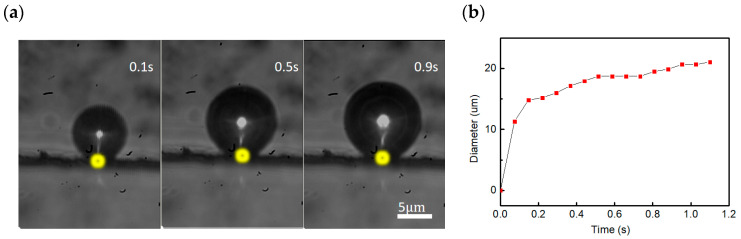
(**a**) Laser-induced microbubble growing in size with laser irradiation time in the soybean oil-filled microchannel. Yellow dots describe the focusing position of the laser beam. (**b**) Diameter–time relationship curve of the microbubble at the oil-CNC interface.

Having established the capability of laser-controlled microbubble motion, we now investigate integrating the laser-controlled motion of microbubbles with microfluidics or functional lab-on-a-chip. Figure 7a shows an optothermally propelled microbubble generation and growth at the interface between soybean oil and the CNC rail attached to the inner wall of the microchannel in a microfluidic chip under laser irradiation within 0.9 s (a laser power of 0.88 mW). The bubble grows rapidly within the initial 0.1 s of laser irradiation, after which its growth rate gradually decreases. At the 1st second of laser irradiation, the microbubble almost does not continue to grow. No obvious change is observed in the bubble diameter with the increase in irradiation time and the microbubble reaches its critical size under laser irradiation within about 1 s, as shown in Figure 7b.

**Figure 8:**
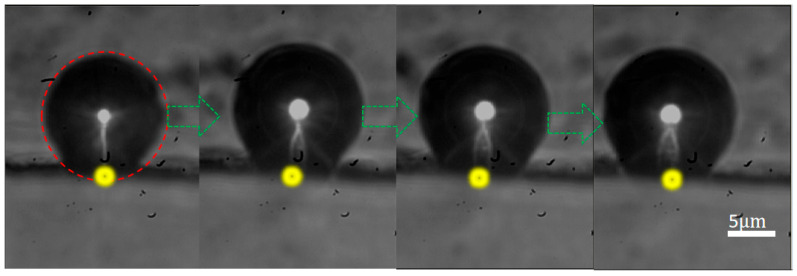
Laser-controlled propulsion of a microbubble following a laser spot on the CNC rail at a steady speed in soybean oil. The green arrows indicate the movement direction of the microbubble on the oil-CNC interface.

Our observations demonstrate that laser-induced microbubbles, when reaching a critical diameter, become capable of tracking the laser spot movement along the oil-CNC rail interface. As shown in Figure 8 (also see Video S3, Supporting Information), a laser-induced microbubble ( 20μm in diameter) in tight contact with the CNC rail rolls forward at 12 μm/s, guided by the manual adjustment of the focus spot along the oil-CNC interface (laser power of 0.88 mW). It is found that propulsion of the laser-induced micro-bubble along the CNC rail is induced only when the microbubble diameter reaches a critical value, 20 μm, at a laser power of 0.88 mW. If the diameter of microbubbles is less than the critical value at a laser power of 0.88 mW, the microbubble fails to undergo laser-guided rolling locomotion along the CNC rail and remains in place. When the power of the laser is below 0.88 mW, no formation of microspheres is observed. When the power of laser exceeds 0.88 mW, a microbubble generates at an excessively high rate, owing to the rapid accumulation of heat on CNCs, making the laser-controlled motion of a microbubble challenging.

**Figure 9:**
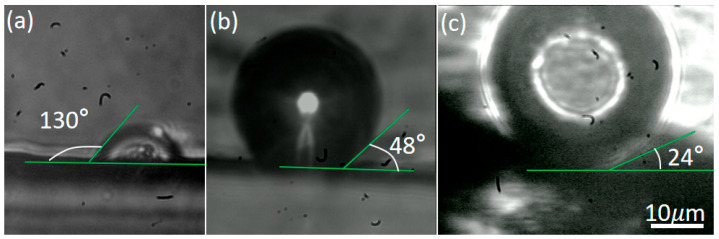
(**a**–**c**) Comparison of microbubble contact angles on CNC rails separately immersed in water, soybean oil and molten paraffin.

The movement speed of microbubbles at the oil-CNC interface is relatively slower than that at the paraffin-CNC interface. To conduct a comparative study, the soybean oil in microchannel is replaced with deionized water, a liquid with a greater surface tension. However, no motion of the microbubbles following the trajectory of the laser spot on the CNC rail is observed after the successful generation of microbubbles at the water-CNC interface, indicating that the laser does not propel the microbubbles’ movement on the CNC rail. The contact angles of microbubbles may be a potential primary factor affecting the movement of microbubbles [56]. Figure 9 shows that the contact angles of optically propelled microbubbles at the water-CNC, oil-CNC, and paraffin-CNC interfaces are 130°, 48° and 24°, respectively. The contact angle is the largest at the water-CNC interface, while the values on the oil-CNC and paraffin-CNC interfaces are similar and significantly smaller. This phenomenon can be explained by Young’s equation, as follows [57,58]:

$$\cos\left(\theta \right)=(\gamma_{SG}{-\gamma }_{SL})/\gamma_{LG},$$

θ is bubble contact angle, $\gamma_{SG}$ is solid-vapor interfacial tension (a fixed value determined by the solid and gas properties). $\gamma_{SL}$ is solid-liquid interfacial tension (varies significantly with the liquid) and $\gamma_{LG}$ is liquid-vapor surface tension. In a water medium, water exhibits high surface tension ($\gamma_{LG}$) and strong interfacial interactions between the water and CNC surfaces [50,59], leading to a comparatively high $\gamma_{SL}$. Since the $\gamma_{SG}$ is determined solely by the solid and gas, the term $\left(\gamma_{SG}\;-\;\gamma_{SL}\right)$ decreases. Consequently, this leads to a smaller value of cos(θ), which corresponds to a larger θ. However, for an oil phase or a molten paraffin wax phase, the liquids exhibit low surface tension ($\gamma_{LG}$) and weak interaction between liquid and CNC, leading to a relatively low value of $\gamma_{SL}$. The term $\left(\gamma_{SG}\;-\;\gamma_{SL}\right)$ increases. Consequently, this leads to a larger value of cos(θ), which corresponds to a smaller θ. Therefore, small contact angles resulting from low surface tension and weak interactions with CNCs are conducive to the microbubbles rolling forward on CNC rails.

When the laser spot deviates from the oil-CNC interface, the rolling microbubble ceases movement without a significant shrinkage in size for a long time in the microchannel. By refocusing the laser beam at the bubble-CNC interface and precisely controlling the laser spot movement along the paraffin-CNC interface, the laser-controlled motion of microbubbles is reinitiated from its quiescent state. Video S3, in the Supplementary Materials, describes the bidirectional motion of a microbubble, which first rolls from left to right and then backs from right to left, by manually adjusting the laser spot. Overall, CNC rails enable the laser-controlled microbubbles to roll reproducibly to a pre-defined location, offering a novel tool for precise manipulation of microbubbles in a microchannel chip.

## 4. Conclusions

We present a new design and method for controlling the propulsion of microbubbles on CNC rails via a dynamic thermal gradient induced by laser irradiation. Owing to the photothermal conversion and surface hydrophobic properties of CNCs, the CNCs serve as thermally powered rails that provide an asymmetric flow field for microbubble propulsion and as support rails that supply the necessary rolling friction for microbubble motion. The propelled microbubbles exhibit forward rolling at the liquid-CNC interface while following the laser spot. The results show that microbubbles can be effectively generated and propelled at the paraffin-CNC interface and the oil-CNC interface by laser irradiation. The movement speed of microbubbles at the oil-CNC interface is comparatively slower than that at the paraffin-CNC interface owing to the difference in contact angle with the CNC rails surface. Additionally, a microbubble can be generated, transported to a desired location, stopped, and re-mobilized repeatedly on demand (without a significant change in volume) within a microfluidic chip by controlling the laser spot. We envision the future use of laser-controlled microbubbles as an on-chip optical element. Microbubbles can be used as a lightbulb positioned on the inner wall of microchannels when incident light undergoes total internal reflection within the microbubble, to provide built-in lateral illumination for enhanced imaging and signal detection of cells or microparticles, eliminating complex external optics.

## Data Availability

Data that support the findings of this study are available from the corresponding authors upon reasonable request.

## Supplementary Materials

The following supporting information can be downloaded at: https://www.mdpi.com/article/10.3390/nano16010005/s1, Video S1: A microbubble rolling forward on the paraffin-CNC interface under laser beam irradiation. Video S2: Flow field around the microbubble on the paraffin-CNC interface. Video S3: Bidirectional motion of microbubble rolling forward on the oil-CNC interface within a microfluidic chip under laser beam irradiation.
